# Student teachers' perceptions of the collaborative relationships form between universities and inclusive elementary schools in Indonesia

**DOI:** 10.12688/f1000research.74999.1

**Published:** 2021-12-16

**Authors:** Rasmitadila Rasmitadila, Megan Asri Humaira, Reza Rachmadtullah

**Affiliations:** 1 Elementary School Teacher Education, Universitas Djuanda, Bogor, Jawa Barat, 16720, Indonesia; 2 Elementary School Teacher Education, Universitas PGRI Adibuana, Surabaya, Jawa Timur, 60234, Indonesia

**Keywords:** inclusive education, collaborative relationship, university, inclusive elementary school

## Abstract

**Background**: The collaborative relationship between universities and inclusive elementary schools has not been maximally practiced. The form of collaboration that universities with inclusive elementary schools have carried out is still limited to the need to complete lecture materials in the semester. There is a gap between the theory and practice obtained by student teachers at universities when they have to teach in inclusive elementary schools. As a result, they have not contributed to solving problems that occur in inclusive elementary schools. The collaborative relationship between inclusive elementary schools and universities directly implies that the success of inclusive education is determined by the competence of student teachers whose universities have successfully educated them in order to teach in inclusive elementary schools. Against the background that the inclusive education system is developing in Indonesia, the researchers investigated student teachers' perceptions at universities about inclusive university-inclusive elementary school collaborative relationships.

**Methods**: During data collection, an online survey and in-depth interviews of student teachers about individual experiences and their ideas about the form of inclusive elementary schools-university partnerships was conducted. The data analysis used is a thematic analysis technique.

**Result**: The result summarizes student teachers' statements, revealed three main themes: provision of inclusive education needs, research, and field practice. The student teachers revealed that the collaborative relationship between universities and inclusive elementary schools is essential to develop holistic, inclusive practices in a collaborative partnership based on input-needs, which has a two-way impact or benefit for both parties.

**Conclusions**: Furthermore, collaborative relationships must be in the form of long-term programs, such as continuous assistance, and adaptation to the development of inclusive education through lecture materials. To achieve inclusive education in Indonesia, we also recommend that the government make policies on multi-sectoral collaboration in order to support inclusive education *.*

## Introduction

The relationship between universities and elementary schools is a positive form of collaboration so that the implementation of education can occur according to the policies set by the government. Several concepts of collaboration, such as the form of partnership between universities and elementary schools, provide benefits for achieving educational goals beneficial for both parties ( Durnan, 2016; Samena et al., 2012). In the context of inclusive education, the collaborative relationship between universities and inclusive elementary schools is expected to increase the quality of inclusive education so that all education stakeholders can experience the results ( Relojo & Pilao, 2018; Zagona et al., 2017).

In the context of Indonesia, the Ministry of Education, Culture, Research, and Technology has issued government regulation Number 13 of 2020, Article 5. It states that to accommodate special needs students (SNSs), universities must organize faculties of education and teacher training, especially study programs or departments of elementary school teachers and said education must provide inclusive education courses. This policy explains a great attachment and responsibility between universities and the competence of student teachers who will teach in inclusive elementary schools. Therefore, there is an opportunity to establish formal collaborative relationships between universities and inclusive elementary schools. As a result, universities and inclusive elementary schools have a direct collaborative relationship in implementing inclusive education.

However, in practice, until now, the collaborative relationship between universities and inclusive elementary schools has not been maximally practiced. In particular, the form of collaboration that universities with inclusive elementary schools have carried out is still limited to the need to complete lecture materials in the semester. In addition, inclusive education courses in elementary school teacher education have been implemented as part of government regulatory policies ( Done & Andrews, 2020; Duncan et al., 2021). The students' portion for further study still does not meet the specific competencies to teach in inclusive classrooms. Furthermore, various forms of collaborative relationships with inclusive elementary schools, such as internships and field practice, have not explicitly led to problem-solving in inclusive classrooms. Meanwhile, collaborative relationships with universities are still positioned as places or locations for research, internships, or field practice for inclusive elementary schools without any follow-up from all these forms of activity ( Sanzo et al., 2011). As a result, they have not contributed to solving problems that occur in inclusive elementary schools. In addition, the reciprocal relationship between universities and inclusive elementary schools that can provide mutual benefits or benefits for each party has not been implemented optimally ( Haines et al., 2015; Messiou & Ainscow, 2020).

The collaborative relationship between universities and inclusive elementary schools must be adapted to the needs and portions appropriately and relevantly ( Salisbury & McGregor, 2002). For this reason, it is necessary to develop forms of collaborative relationships that are expected to provide mutual benefits. A collaborative relationship involves interaction between two or more people to achieve a common goal, and is based on trust, respect, and shared responsibility (Cook & Friend, 2010). There are seven essential points involved in developing collaborative relationships: voluntary participation, creating shared goals, sharing resources, shared responsibility for crucial decisions, shared accountability for results, equal parity or contribution of all participants, and the trust and respect that arises (Friend et al., 1993). Moreover, the forms of collaborative relationships that are developed must be carried out for the long-term and meet sustainable interests ( Florini & Pauli, 2018). Therefore, this study aims to explore student teacher's opinions on the form of collaborative relationships between universities and inclusive elementary schools in Indonesia

## Methods

### Research design

This study design consists of simple research design. Simple reseacrh design reflecting experience in the field, using theory to solve problems in the field, with a rigorous literature review, using research instruments, collecting data continuously (surveys and interviews) on a topic related to the problems found ( Bungin, 2020). The steps conducted in this research consist of five steps: first, the researcher chooses a social context, and builds appropriate research questions; second, conduct a literature review to answer research questions; third, data collection using online surveys and interviews; fourth, data analysis using thematic analysis to generate the main themes; Fifth, make a systematic research report. In this study, the research is focused on the form of collaborative relationships between inclusive elementary schools. A literature search was conducted at the beginning of the study to determine the conceptual and operational definitions of the research focus, namely data relating to the form of collaboration between universities and inclusive elementary schools. Consequently, the data collection instrument was based on operational definitions with the main theme of collaboration between inclusive elementary schools.

### Participants

Participants in this study were totally 51 student teachers (STs) from four universities with teaching faculties and elementary school teacher education programs in Jakarta, West Java, Central Java, and East Java in Indonesia. The selection of the four provinces was based on areas with the largest number of elementary schools in Indonesia and had universities with elementary school teacher education. The criteria for STs involved in this study include students who have attended lectures at least in semester 7 (3.5 years), received inclusive education courses, and participated in internships. Through courses and internships, it is hoped that the stundent teacher can explain more deeply about the problems that occur in inclusive elementary schools, and consider the need for collaborative relationships between universities and inclusive elementary schoolsA purposive sampling technique was used to distribute online questionnaires using Google Forms to representatives of research colleagues who have access to universities in the four provinces.

### Data collection

Data collection was conducted in two stages. The first stage was to collect data from STs with the criteria explained in the participant section, using an online survey consisting of three main questions, in an essay--open-ended questions--using Google Forms. The questions were compiled based on the literature relevant to the research objectives. Data were collected from July 25, 2021 - July 29, 2021, and involved 51 respondents.

The collected data, a transcript of the results of each respondent, was made to construct the initial code. The second was semi-structured interviews with 5 of the 51 STs who responded to the survey. Researchers developed interview guidelines based on the initial code from the first stage. The purpose of the interview was to obtain more in-depth and meaningful data related to the research focus. Interviews were conducted for about 1 hour per respondent through the WhatsApp application from August 1, 2021, to August 3, 2021.

To access all respondents in data collection, we obtained permission from lecturers of inclusive education courses from four universities that have held inclusive education courses in the Elementary School Teacher Education Department in Jakarta, West Java, East Java, and Central Java. We made a permit, and they agreed to carry out the data collection process in the form of surveys and interviews. They provided their verbal and written consent due to the good relationship between the researcher and the Head of the Elementary School Teacher Education Departments at the four Universities. Survey responses and interviews are kept confidential to ensure there is no conflict of interest in this study

### Instruments

The instruments used in the two stages of the study consisted of open-ended questions. The instrument in the form of an essay in Google Forms consisted of three open-ended questions. This essay was in the form of a question asking STs to express their ideas or opinions related to university collaboration with inclusive elementary schools based on the experience of inclusive practice that they had undergone thus far. Here are examples of questions on Google Forms provided;

(1) Do you think it is important to have a cooperative relationship between the University/Faculty (Teachers) and inclusive elementary schools? Give your reasons
(2) What do you think the form of relationship or collaboration? Give examples such as teaching experiments or visits to inclusive schools
(3) What do you think are the benefits for the university regarding the relationship or collaboration with the inclusive elementary school? Give reasons

Meanwhile, we used three open-ended questions for the interview instrument focusing on deepening the questions on the results of the STs’ opinions during the first stage. We directed this interview to explore further the needs of inclusive education, research, and field practice. Here we provide examples of interview questions:

(1) What needs for inclusive education are urgently needed in order to build collaboration between universities and inclusive elementary schools?
(2) Why is research so important in collaboration between universities and inclusive elementary schools?
(3) What field practice programs can universities run that benefit STs in supporting preparation to become inclusive teachers in inclusive elementary schools?

### Data analysis

The data analysis used is a thematic analysis technique to identify, evaluate and create the main themes that have been revealed by the researcher ( Braun & Clarke, 2012; Galloway & Jenkins, 2009). In the first stage, opinions from special STs were given a certain code in the form of keywords that match the interpretation of the sentence or certain terms so as not to overlap. Second, researchers used the NVivo 12 program to facilitate coding and categories. Third, the researcher analyzed all codes and categories that allowed for merging and even splitting codes to become simpler codes and could answer research questions in the main themes.

The researcher also considered the credibility and dependability of the data and started from the data collection instrument used based on the relevant literature review. The instrument was research prepared by involving inclusive education experts. After the data was collected, member-checking ( Lincoln, 1985) was also performed, which was used to check the credibility of the participants (especially when interviewing 5 STs). They were asked to clarify that their contribution was accurately reflected in the data. Meanwhile, researcher triangulation was also conducted to reduce bias by cross-examining participants ( Anney, 2014). Thus, the involvement of all researchers in examining data with the same problem would support the integrity of the findings.

### Ethical consideration

The research and community service institute of Universitas Djuanda has approved this research with the contract number: 143/LPPM/K-X/VII/2021. A letter of approval has also been given by the researcher to all respondents. Written consent to participate from the respondent was obtained in accordance with document 280/LPPM/K-X/XI/2021. Respondents have given their consent without force from anyone. Subsequently, in order to protect the rights and privacy of the respondents, all forms of data were acquired will remain confidential.

## Result and discussion

### Result

**Figure 1.**
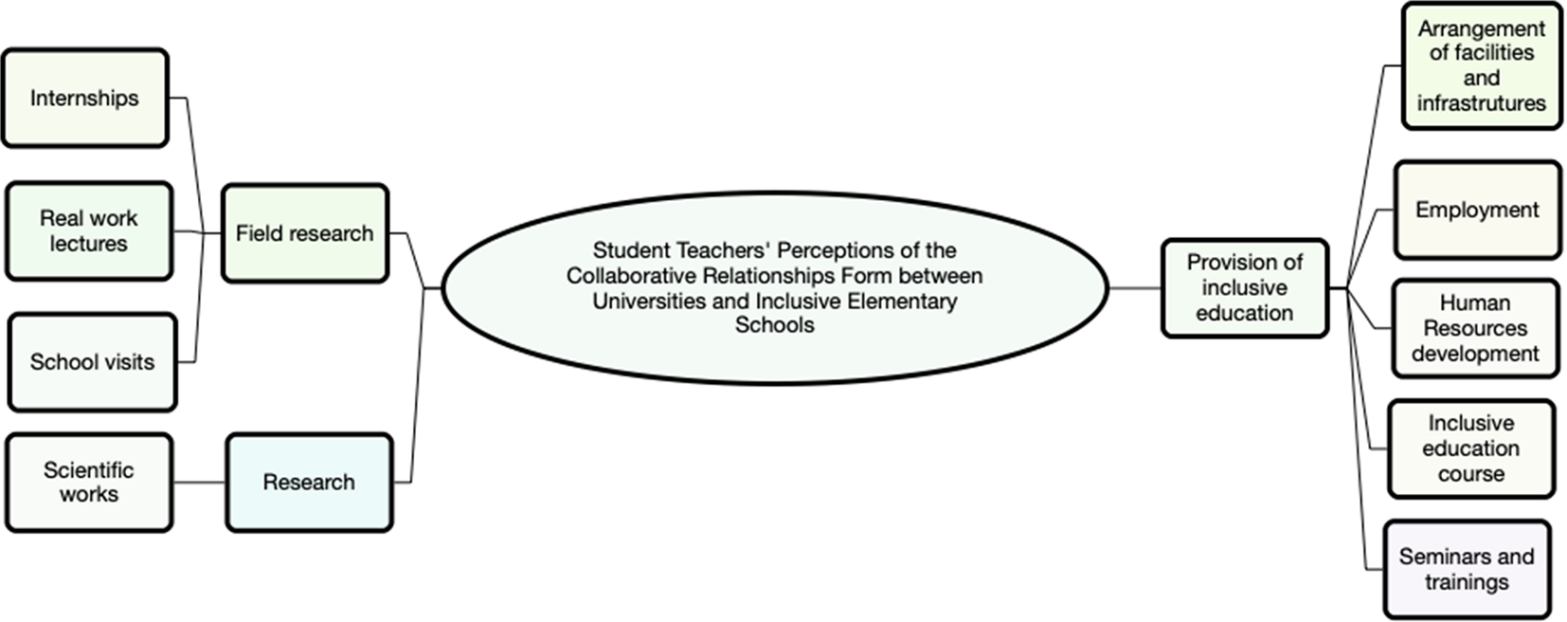
The main themes of thematic analysis (use Nvivo 12).

Thematic analysis revealed three main themes, namely (1) provision of inclusive education needs, (2) research, and (3) field practice. All themes are summarized in Figure 1.

**Provision of inclusive education needs**

The researhers identified five sub-themes related to the main theme, including employment, inclusive education courses, seminars and training, arrangement of facilities and infrastructure, and human resource development. First, the sub-theme of employment relates to the relationship between teacher graduates who will work in inclusive elementary schools. STs hope that the collaboration between universities and inclusive elementary schools will allow graduates to work in inclusive elementary schools. The internship experience or field practice in inclusive elementary schools while still in university provides valuable assessments for inclusive elementary schools to accept teacher graduates to join and teach well. The following statement taken from an ST illustrates this perspective:

[…] if there is collaboration, it will make it easier for me to get a job to teach in inclusive elementary schools. With programs such as internships in inclusive elementary schools, schools will see how the quality of prospective graduates is, and it is not difficult to find new teachers if the school needs a teacher. (ST 4)

Second, the sub-theme of courses is inclusive education related to the importance of the inclusive education's course that must be carried out by study programs of the elementary school teacher education. As a study program that produces inclusive elementary school teachers who teach in inclusive elementary schools, STs hopes that every student teacher must deeply understand inclusive education so that it can be practiced in inclusive elementary schools. STs received information related to the importance of inclusive education courses from previous graduates. By receiving material from inclusive education courses, they could at least solve problems in inclusive classrooms. However, training activities must be conducted continuously by every inclusive teacher. The following statement was taken from an ST to illustrate this idea:

With the course’s inclusion of inclusive education, I hope to practice inclusive education when I later teach in inclusive elementary schools and solve problems in inclusive classes. (ST 35)

Third, the sub-theme of seminars and training provides an overview of the importance of seminars and continuous training for inclusive elementray school teachers, organized by the faculty or even the university level. The faculty has many experts in the field of inclusive education to organize scheduled seminars and training according to the topics or problems faced by inclusive elementary schools. Problems that occur in inclusive classrooms must be solved as to positively impact the practice of inclusive education. Several STs explained this opinion:

One form of collaborative relationship that universities can provide is to provide seminars for inclusive teachers. (ST 2)

Collaboration provides training on the science of forms of instructional strategies, instructional tools, instructional media, and other supporting tools. (ST 5 & ST 17)

Fourth, the sub-theme of structuring learning facilities and infrastructure is important in fulfilling the need for inclusive practice. The form of collaborative relationship expected by STs is the provision of good physical facilities needed by inclusive elementary schools, which are still very limited. STs hope that inclusive elementary schools that receive children with special needs such as those who are blind, quadriplegic, or those who require special physical treatment, can have their rights fulfilled in inclusive schools. Limited funding for inclusive elementary schools in the provision of infrastructure is still a big problem that universities must solve. In addition, the availability of instructional media or instructional support tools that teachers will use to teach special needs students still requires great improvement. A statement from one of the ST:

Universities must help inclusive schools, especially the provision of facilities and infrastructure such as wheelchairs for students with disabilities, learning media for blind students (Braille), or other learning media developed by universities. (ST 18)

Fifth, the sub-theme of human resource development is related to improving all human resources' competence that supports the implementation of inclusive education in inclusive elementary schools. Human resources consist of all members of the inclusive elementary school community (e.g., principals, teachers, staff), including STs who will become teachers in inclusive elementary schools. In addition, lecturers who teach inclusive education courses in teacher education study programs also require self-development to keep up with the latest developments related to research results in the field of inclusive education, which has an impact on the novelty of lecture material for STs. One of the STs gave his opinion:

In my opinion, human resource development is critical, especially for all members of inclusive schools, to keep up with changes in inclusive education. This development can be aided by study programs at universities, through providing training. (ST 21)

**Research**

The research theme produces sub-themes related to scientific works, which results from collaboration between universities and inclusive elementary schools. From the STs’ perspective, inclusive elementary schools are the most frequently used places for research activities. Lecturers, classroom teachers, and STs can collaborate in mapping the problems they face (e.g., instructional methods, instructional media, etc.) in order to further conduct research to provide solutions to these problems. Success in research with this collaboration must be able to be disseminated to other inclusive schools in the form of scientific work and is expected to impact inclusive practice. Several STs explained this opinion in expressing the need for research:

[…] it would be very good to collaborate with inclusive elementary schools by conducting joint research between lecturers, students, and inclusive teachers. (ST 5)

Students and inclusive teachers can produce scientific papers from research whose results can be used by other inclusive schools, and very helpful in solving problems that have been ocurring in inclusive schools. (ST 6)

**Field practice**

The sub-theme of field practice explains real work lectures, school visits, and internships. The sub-theme of real work lectures is a form of collaboration that provides opportunities for STs to be closer to the community in practicing and applying the science learned from lectures in the community. Real work lecture activities, especially in the field of education in areas that are not yet developed (rural) and the quality of education is still low, are expected to make an impact, especially on spreading awareness of inclusive practices. Students and field supervisors can provide real examples of inclusive practices, both in academic and non-academic aspects. The following examples taken from one ST illustrate the need for collaboration between universities and inclusive elementary schools related to real work lectures: real work lectures in villages, especially those with inclusive elementary schools, will provide good benefits for the inclusive elementary schools because many schools in villages do not understand inclusive education.

The sub-theme of visits to inclusive elementary schools is a form of collaborative activity relationship that both parties can continuously carry out. The visit to the school aims to find information and map the problems of inclusive practice that inclusive teachers often face. According to STs, visits to inclusive schools can be an input for universities to update inclusive education lecture materials so that they are more relevant to the problems faced by inclusive elementary schools. An example of one ST's opinion can be illustrated in the statement below:

I think that a visit to an inclusive elementary school is beneficial so that universities get positive input, especially for the improvement and renewal of inclusive education courses. (ST 25)

The sub-theme of internship relates to the opportunity given to STs to try teaching in inclusive elementary schools as a way to gain teaching experience that will be used when becoming an inclusive teacher. The collaborative relationship between universities and inclusive elementary schools is carried out by sending STs to inclusive elementary schools to perform their duties and responsibilities as prospective teachers in inclusive schools. Internship activities such as assisting teachers in learning in inclusive classrooms, class management, and class administration will provide work experience and situations that STs will face when they graduate from university. STs will practice teaching in the classroom with real conditions, solving problems directly with the classroom teacher. It will produce prospective inclusive teachers who are intentional in the future. One of the STs stated this during internship:

Universities can send their students to inclusive elementary schools to teach there so that students obtain real experience in teaching; together with teachers, they can solve problems in inclusive classrooms. (ST 17)

### Discussion

In this study, the researchers conducted an online survey, interviewed student teachers from universities with study program of elementary school teacher education who are prospective teachers who will teach in inclusive elementary schools in Indonesia. The researchers asked questions related to the form of collaboration between universities and inclusive primary schools. Student-teacher statements were analyzed against the theoretical background and research findings in collaborative relations between universities and inclusive primary schools. The use of thematic analysis provided an opportunity for the researchers to investigate the further form of universities and inclusive elementary school collaboration in all aspects of inclusive education delivery.

The reseachers identified several themes and sub-themes that reflect student teachers' opinions about the forms of collaboration that can be carried out between universities and inclusive elementary schools in Indonesia. Although the Indonesian government has made a policy to develop inclusive schools through the ministry of education and culture, every educational study program for students at universities must provide inclusive education courses as well. However, the impact of this policy only provides initial understanding to students. Therefore, university collaboration through study programs is still one-way, especially in fulfilling the obligations of completing student studies and the study program itself ( Becht et al., 2020). There are several forms of collaborative relationships between universities and elementary schools in programs ( Norris & Martin, 2021) such as internships and student research--completion of final assignments--but there is no specific collaboration with inclusive elementary schools, whose programs are also specifically directed to solving problems of inclusive practice ( Passy et al., 2018). For example, the new inclusive elementary school is an inclusive education laboratory that provides opportunities for every student teacher to gain teaching experience in inclusive classrooms and gain a real picture of inclusive education problems. But unfortunately, universities' study programs have not followed up on these problems as a form of reciprocal collaboration that should be a source of problem-solving for inclusive elementary schools ( Pollock & Briscoe, 2019).

Despite there being many research results obtained by universities related to inclusive education in inclusive elementary schools, the continuation and benefits of these research results have not been felt by inclusive elementary schools. The reason is that the collaborative relationship between universities and inclusive elementary schools is still one-way, which tends to benefit the university, especially academically ( Miller et al., 2017; Guadarrama et al., 2008). Therefore, it is necessary to change to the form of a two-way collaborative relationship that can help both parties, which is based on needs-input as a common way to solve inclusive education problems in elementary schools ( Derzhavina et al., 2021; Kurth et al., 2020). Universities and inclusive elementary schools must be able to translate the needs of inclusive education together. This form of two-way collaboration provides opportunities and benefits to schools, such as increasing teacher competence through seminars or training programs organized by universities ( Steel et al., 2019; Lynch, 2013) on an ongoing basis and joint procurement of inclusive school facilities and infrastructure. For universities, the results of research conducted by lecturers and students and results from student field practice are inputs needed to meet the needs of student teachers ( Demirel et al., 2018). Furthermore, universities can design inclusive education courses based on empirical research results so that the relevance of courses-theoretically-with practice in inclusive elementary schools can be used to solve problems that inclusive elementary schools face ( Zelina, 2020; Benade, 2019). The researchers believe that the results of this research can change and develop forms of collaborative relations between universities and inclusive elementary schools, which so far have not provided two-way benefits to both parties, and can be adapted to the development of inclusive education. In general, the researchers hope that the results of this study can contribute to the overall advancement of inclusive education in Indonesia.

### Limitation

The limitations of this study are the determination of criteria that require that student teachers have received inclusive education courses, and have conducted internships, especially in inclusive schools from the elementary school teacher education department. Meanwhile, not all elementary school teacher department receive inclusive education courses, so the number of respondents is still limited.

## Conclusion

Collaborative relationships between universities and inclusive elementary schools are needed to develop holistic and inclusive practices. This relationship must be based on collaborative partnerships, which has a two-way impact or benefit for both parties. The form of collaborative relations is considered based on mutual input and needs that are practiced in real programs as part of the development of inclusive education in Indonesia. The researchers recommend the results of this research to inclusive universities and elementary schools in order to establish collaborative relationships in the form of long-term programs, such as continuous or sustainable mentoring, and adaptation to the development of inclusive education through lecture materials. The researchers also recommend the government makes policies on multi-sectoral collaboration in supporting inclusive education.

Besides, there is a need for further research related to the model of sustainable and sustainable mentoring programs based on collaborative partnerships between universities and inclusive elementary schools.

## Competing interests

No competing interests were disclosed.

## Grant information

This study was supported by Directorate of Resources; Directorate General of Higher Education; Ministry of Education, Culture, Research and Technology of the Republic of Indonesia with Higher Education Service Institutions-Regional IV and Research and Community Service of Universitas Djuanda with Agreement/contract Number: 310/SP2H/LT/DRPM/2021, March 18, 2021;016/SP2H/RDPKR-JAMAK/LL4/2021 dated 19 July 2021; 143/LPPM/K-X/VII/2021 dated July 21, 2021. The head of research grant is Rasmitadila Rasmitadila.

The funders had no role in study design, data collection and analysis, decision to publish, or preparation of the manuscript *.*

## Data availability

Figshare. Data of Questioner and Interview-Rasmitadila.docx. DOI: https://doi.org/10.6084/m9.figshare.16782553.v1

This project contains the following underlying data: - Dataset of Questioner and Interview from the participants.

Data are available under the terms of the Creative Commons Attribution 4.0 International license (CC-BY 4.0).
