# Supplementary material for: Student teachers' perceptions of the collaborative relationships between universities and inclusive elementary schools in Indonesia
Source: F1000Res. 2022 Sep 16;10:1289. Originally published 2021 Dec 16. [Version 4] doi: 10.12688/f1000research.74999.4 (PMC9437534; doi:10.12688/f1000research.74999.4)
Supplement: Peer review history

# Peer review: Reviewer response for version 3

**DOI:** 10.5256/f1000research.136926.r146894

**Published:** 2022-09-01

**Authors:** Mirna Nel

**Affiliations:** 1 Faculty of Humanities, North West University, Vanderbijlpark, South Africa

Please revise the following sentence:

 " *Regarding the research aims, the main of research questions is “how are the forms of collaborative partnerships between universities and inclusive elementary schools in Indonesia?”. To the main focus in more detail on this research question, we created specific questions (see examples of questions on page 6).*"

 Change to:

  The main research question was: “how are the forms of collaborative partnerships between universities and inclusive elementary schools in Indonesia?”. To put the main focus of the research question in more detail secondary questions were developed (see examples of questions on page 6). 

 It should not be examples - it must be the questions that were used to guide the focus and implementation of the research and that are addressed and answered in the article.

 Please replace "still" in the following text  - the word is used too much

- "...quasi-qualitative is a part of research that is still influenced by the influence of positivism which is still used in the presentation of theory, which is still deductive, so this research cannot be said to be fully qualitative. Still, it can be seen during the process of analyzing the data..."
- There is still an inconsistent use of researcher and researchers. Since there are three authors I assume plural researchers were involved in the research study?
  "Thus, the involvement of all the researchers in examining data with the same problem would supported the integrity of the findings" Please fix language
  The researcher gave a letter of approval has also been given by the researcher to all respondents.. Please fix the language 

Unfortunately, there are still many such basic language mistakes throughout the article. I fully understand that English is not the first language of the authors, but for publication it is important to demonstrate a good scholarly language ability. I do not have a problem with the application of the research and the consequent findings. It makes a good contribution to the field.

Is the work clearly and accurately presented and does it cite the current literature?

Partly

If applicable, is the statistical analysis and its interpretation appropriate?

Not applicable

Are all the source data underlying the results available to ensure full reproducibility?

Yes

Is the study design appropriate and is the work technically sound?

Partly

Are the conclusions drawn adequately supported by the results?

No

Are sufficient details of methods and analysis provided to allow replication by others?

Partly

Reviewer Expertise:

Inclusive education

I confirm that I have read this submission and believe that I have an appropriate level of expertise to confirm that it is of an acceptable scientific standard.

# Response

**Published:** 2022-09-08

**Authors:** Rasmitadila Rasmitadila

**Affiliations:** Universitas Djuanda, Indonesia

Dear Prof. Mirna Nel,

 We thank to you for the evaluation of our work and appreciate the positive comments. We have made several revisions such as the suggestions you have given us. Some of the words "still" we have adjusted to the sentence. We also try to improve other words, to match the quality of the language understood by international readers.

# Peer review: Reviewer response for version 3

**DOI:** 10.5256/f1000research.136926.r146893

**Published:** 2022-08-18

**Authors:** Urip Sulistiyo

**Affiliations:** 1 Universitas Jambi, Jambi, Indonesia

It has been revised as advised. Thanks

Is the work clearly and accurately presented and does it cite the current literature?

Yes

If applicable, is the statistical analysis and its interpretation appropriate?

Not applicable

Are all the source data underlying the results available to ensure full reproducibility?

Partly

Is the study design appropriate and is the work technically sound?

Yes

Are the conclusions drawn adequately supported by the results?

Yes

Are sufficient details of methods and analysis provided to allow replication by others?

Partly

Reviewer Expertise:

Teacher Education, teacher professional development, and language teaching

I confirm that I have read this submission and believe that I have an appropriate level of expertise to confirm that it is of an acceptable scientific standard.

# Peer review: Reviewer response for version 2

**DOI:** 10.5256/f1000research.134096.r138382

**Published:** 2022-07-18

**Authors:** Mirna Nel

**Affiliations:** 1 Faculty of Humanities, North West University, Vanderbijlpark, South Africa

This article has been appropriately improved with regard to methodology. However, there are still some basic language issues that must be addressed throughout. For example:

- P3 the research questions is: there is only one question
- Look at the appropriate use of "the": Please use "the researchers" and not only researchers when reference is made to the researchers of this study
- Page 5. "present inclusive education courses" not " held inclusive education courses" 
- Although the language has improved it is really important to do another thorough language edit.
- Not sure what it means " to make a permit"?
- Confidentiality is not applicable to only conflict of interest, but is much broader. Having a good relationship with an institution to get permission for data collection can be deemed an ethical issue.

Is the work clearly and accurately presented and does it cite the current literature?

Partly

If applicable, is the statistical analysis and its interpretation appropriate?

Not applicable

Are all the source data underlying the results available to ensure full reproducibility?

Yes

Is the study design appropriate and is the work technically sound?

Partly

Are the conclusions drawn adequately supported by the results?

No

Are sufficient details of methods and analysis provided to allow replication by others?

Partly

Reviewer Expertise:

Inclusive education

I confirm that I have read this submission and believe that I have an appropriate level of expertise to confirm that it is of an acceptable scientific standard, however I have significant reservations, as outlined above.

# Response

**Published:** 2022-07-31

**Authors:** Rasmitadila Rasmitadila

**Affiliations:** Universitas Djuanda, Indonesia

Dear Prof. Mirna Nel,

 We thank to you for the evaluation of our work and appreciate the positive comments. Please see below, for a point-by-point response to the reviewers’ comments:

 1. We have added information related to some examples of research questions (on page 6) so that readers can immediately check some research questions that can answer the main research questions.

 2. We have also revised and added some words according to suggestions, such as "the" in the researcher; "present inclusive education courses,"; and the word "We made a permit" to "We asked permission from the lecturers." This statement explains that before we collect data, we ask the lecturers for permission regarding their consent to become participants in our research. If they agree, we will collect data (interview) with them.

 3. We have completely re-edited the language for the whole article

# Peer review: Reviewer response for version 2

**DOI:** 10.5256/f1000research.134096.r138383

**Published:** 2022-07-06

**Authors:** Urip Sulistiyo

**Affiliations:** 1 Universitas Jambi, Jambi, Indonesia

I have read the revision of the manuscript and I have no further comments to make as the authors have revised all necessary revision issues of the paper.

Is the work clearly and accurately presented and does it cite the current literature?

Yes

If applicable, is the statistical analysis and its interpretation appropriate?

Not applicable

Are all the source data underlying the results available to ensure full reproducibility?

Partly

Is the study design appropriate and is the work technically sound?

Yes

Are the conclusions drawn adequately supported by the results?

Yes

Are sufficient details of methods and analysis provided to allow replication by others?

Partly

Reviewer Expertise:

Teacher Education, teacher professional development, and language teaching

I confirm that I have read this submission and believe that I have an appropriate level of expertise to confirm that it is of an acceptable scientific standard.

# Response

**Published:** 2022-07-30

**Authors:** Rasmitadila Rasmitadila

**Affiliations:** Universitas Djuanda, Indonesia

Dear Mr. Urip Sulistiyo,

 We thank to you for the evaluation of our work and appreciate the positive comments.

# Peer review: Reviewer response for version 1

**DOI:** 10.5256/f1000research.78810.r126822

**Published:** 2022-04-06

**Authors:** Mirna Nel

**Affiliations:** 1 Faculty of Humanities, North West University, Vanderbijlpark, South Africa

The article has potential. It appears as if important research has been done that could contribute to teacher education which ensures that student teachers are adequately prepared for inclusive education.

- Unfortunately, this was an incoherent report of the research done. First of all it needs a thorough language editing with regard to sentence construction, concept definitions and the use of the correct terms in the context of the statements.
- Past tense should be used for research which is reported in an article as it is already conducted.
- The title needs re-thinking. Maybe leave out "form".
- There is not a clear literature review which substantiates a distinct problem statement. I could also not find the research question/s.
- Maybe add a description of what an inclusive elementary school entails.
- This was a qualitative study and it is nowhere stated. In a qualitative study, it is recommended that participants are used rather than respondents, but these two terms are used intermittently. 
- There is no clear description of the sampling technique.
- Because there is not a distinctive problem statement and research questions, the link between the survey and interview questions with the focus of the research is not clearly aligned. This also results in the findings and discussion needing better construction to answer the research aim.
- With open questions there should not be a yes/no question such as "do you think..."
- This question does not make sense: 'What do you think the form of relationship or collaboration?'

If the article is adapted to provide a better coherent description of the research it has potential to be indexed.

Is the work clearly and accurately presented and does it cite the current literature?

Partly

If applicable, is the statistical analysis and its interpretation appropriate?

Not applicable

Are all the source data underlying the results available to ensure full reproducibility?

Yes

Is the study design appropriate and is the work technically sound?

Partly

Are the conclusions drawn adequately supported by the results?

No

Are sufficient details of methods and analysis provided to allow replication by others?

Partly

Reviewer Expertise:

Inclusive education

I confirm that I have read this submission and believe that I have an appropriate level of expertise to state that I do not consider it to be of an acceptable scientific standard, for reasons outlined above.

# Response

**Published:** 2022-05-16

**Authors:** Rasmitadila Rasmitadila

**Affiliations:** Universitas Djuanda, Indonesia

Dear Prof. Mirna Nel,

 Thank you for allowing us the opportunity to submit a revised draft of the manuscript “ Student teachers' perceptions of the collaborative relationships form between universities and inclusive elementary schools in Indonesia” for publication in the F1000Research.

 We appreciate the time and effort that you dedicated to providing feedback on our manuscript and are grateful for the insightful comments and valuable improvements to our paper. We have incorporated most of the suggestions made by the reviewers. Please see below, for a point-by-point response to the reviewers’ comments and concerns:

 We have done some sentence editing using a proofreader (native) to improve the language in this article. For the title, we have made improvements by removing the word "form" so that it becomes better.

 We have also added research questions, including a description of what inclusive primary schools are. For the methods section, especially in the research approach, this is a quasi-qualitative study (the explanation is in the article), we use the word respondent (for the initial step), and in the second step (interview), we use participants related to the interview process.

 For the questions in the questionnaire, we don't just use words with yes or no answers. Still, we ask respondents to give their reasons so that we get written answers that can also represent the respondent's thoughts, which we then elaborate on in the interview. But to make it clearer and more focused, in this revision, we have changed the form of the question with a sentence that leads to an open answer that allows us to answer the purpose or research question.

 Thank you so much.

 Best regards,

 Rasmitadila, Rasmitadila

# Peer review: Reviewer response for version 1

**DOI:** 10.5256/f1000research.78810.r119531

**Published:** 2022-02-07

**Authors:** Urip Sulistiyo

**Affiliations:** 1 Universitas Jambi, Jambi, Indonesia

Dear Authors,

 I have carefully read and reviewed the manuscript. The design and its writing are technically sound. However, several issues need to be addressed before it is suitable for indexing:

1. As the authors used purposive sampling technique, it is necessary to provide a table showing demographic information of the participants and provide reasons of nominating 5 out of 51 student teachers.
2. The author needs to provide a table showing the results of thematic analysis generating from NVivo 12 program.
3. Since the data are from open ended survey and interview, please provide details in a way the authors employed those two data both in data analysis and discussion.

Thank you, and good luck.

Is the work clearly and accurately presented and does it cite the current literature?

Yes

If applicable, is the statistical analysis and its interpretation appropriate?

Not applicable

Are all the source data underlying the results available to ensure full reproducibility?

Partly

Is the study design appropriate and is the work technically sound?

Yes

Are the conclusions drawn adequately supported by the results?

Yes

Are sufficient details of methods and analysis provided to allow replication by others?

Partly

Reviewer Expertise:

Teacher Education, teacher professional development, and language teaching

I confirm that I have read this submission and believe that I have an appropriate level of expertise to confirm that it is of an acceptable scientific standard, however I have significant reservations, as outlined above.

# Response

**Published:** 2022-05-16

**Authors:** Rasmitadila Rasmitadila

**Affiliations:** Universitas Djuanda, Indonesia

Dear Mr. Urip Sulistiyo,

 Thank you for allowing us the opportunity to submit a revised draft of the manuscript “ Student teachers' perceptions of the collaborative relationships form between universities and inclusive elementary schools in Indonesia” for publication in the F1000Research.

 We appreciate the time and effort that you dedicated to providing feedback on our manuscript and are grateful for the insightful comments and valuable improvements to our paper. We have incorporated most of the suggestions made by the reviewers. Please see below, for a point-by-point response to the reviewers’ comments and concerns: 

 1) Figure 1 is the result of data analysis using Nvivo 12. We use a mind map type of display data generated in Nvivo 12 (not a table) but have the concept of data analysis results between the two.

 2) The data collected from the Google Form, which is the result of the survey in the first step, and the form of a transcript of the results of each respondent, is made to compile the initial code. This initial code is used to make questions for interviews with selected participants. So the second step is semi-structured interviews with 5 of the 51 STs who responded to the survey. Researchers develop interview guidelines based on the initial code from the first stage. The purpose of the interview is to get a more in-depth and meaningful picture of data related to the research focus. The results of the interviews are entered back into the Nvivo system as Nodes and compiled with the results of previous surveys. Researchers have found a theme that fits the research objectives. Interviews were conducted for approximately 1 hour per respondent via WhatsApp applications from August 1, 2021, to August 3, 2021.

 3) We have added the profile of the participants (demography) in the section on participant
